# Supplementary material for: The role of chromatin state in intron retention: A case study in leveraging large scale deep learning models
Source: PLoS Comput Biol. 2025 Jan 10;21(1):e1012755. doi: 10.1371/journal.pcbi.1012755 (PMC11756788; doi:10.1371/journal.pcbi.1012755)
Supplement: Attachment — Submitted filename: reviewer_response.pdf [file pcbi.1012755.s001.pdf]

The role of chromatin state in intron retention: a case study in leveraging large scale deep learning models

Response to reviewer comments

We thank the reviewers for their detailed comments. Here is a summary of the changes since the initial review:

- Additional paragraph in the introduction comparing our work with other ML work related to splicing.
- Additional paragraph in the results/discussion section to provide additional biological context to our findings on the role of transcription factors in intron retention.
- An additional table in the results section that provides a summary of the performance of all the methods discussed in the manuscript. For completeness, we compare the performance of our fine-tuning approach with a simpler approach that simply replaces the classification block. This worked almost equally as well as the fine-tuning block with an additional convolutional layer.
- The GitHub repository was revamped, addressing errors that affected the reproducibility of the results.

Reviewer 1:

- The paper's argument for the use of foundation models in genomic contexts because of the complexity of the genome is interesting. The authors make the case specifically for leveraging a chromatin state foundation model for prediction of intron retention and compare its merits against other deep learning models.

We think so too!

- Considering that the paper is framed as a case study, I think it would be enhanced by further discussion of the biological motivation for the work.

We expanded the discussion of the biological motivation in the introduction; we also added a new paragraph in the results section that discusses the biological context of our findings as follows:

How do TFs regulate IR?
The results of the Sei-targets model based on TFs suggest that regulation of IR is coordinated by a large number of TFs. While the literature contains ample discussion of the connection of the chromatin landscape and splicing~\cite{yustis2024functional}, there

are only isolated examples of TFs associated with IR, and their role as a whole has not been widely explored. There are several ways in which chromatin state has been proposed to affect splicing, and TFs fit well within these mechanisms. In the \emph{kinetic model}, the speed of Pol II elongation modulates the availability of splice sites thereby affecting splice site choice~\cite{yustis2024functional}. TFs, through their effect on chromatin architecture, are capable of orchestrating elongation rates either directly, or through their effect on histone modifications and other factors that affect nucleosome positioning. An alternative way in which TFs can affect splice site choice is through the \emph{recruitment model}: We propose that like the CTD domain of Pol II \cite{yustis2024functional}, TFs can interact with splicing factors and function to orchestrate their co-transcriptional recruitment. Further experimental work is required to explore these hypotheses.

- In Section 2, the paper would benefit from further clarification/discussion of the occurrence of duplicates in the ranked list of DNA binding proteins and how that affects the analysis of results.

This is discussed in two separate locations in the manuscript:

"…a given DNA binding protein can occur multiple times on the list, as a given protein might have been assayed in different cell lines or tissues and therefore associated with multiple Sei targets. And indeed, among the 9,471 DNA-binding targets there are only about 1,100 unique proteins. As a result, a given DNA binding protein will be assigned multiple ranks, reflecting the different activities or levels of activity that a protein can have in different tissues or conditions."

Later in the manuscript we also mention that a given TF can occur both as contributing to IR and constitutive splicing by having occurrences with both positive and negative weights:

"We observed that some TFs have both large positive weights and large negative weights, indicating that a given TF can both promote IR and constitutive splicing in different cellular contexts. The top 100 and bottom 100 ranked TFs had an intersection of 22 TFs that include CTCF, BRD4, POLR2A, and MYC. As mentioned above, this is possible since a given TF can be represented in multiple Sei targets describing its activity in different tissues or cell lines."

Please let us know if you require additional clarification.

Code review.

We thank the reviewer for the detailed comments on the code provided in the GitHub repository. We apologize for several errors. The repository has been revamped and verified ahead of the resubmission, and the results should now be consistent with what's in the manuscript. Some details of the issues raised by the reviewer:

Reproducibility

- I was unable to produce results for either the LR, LGBM, or FDNABert models using the code and instructions provided. See errors list below for details.

For the LR and LGBM models, in the code in the GitHub repository there was an extra padding parameter (pad the sequences from 600 bps to 4kb) that was used in the Python script that generates the numpy input files. In our results we didn't use that parameter, which made the Sei predictions slightly different, yielding different results.

We had a wrong import statement in FDNABert.py as you had indicated.

- Running the ConvNet (called Bassenji-like in the manuscript?) and Basset models with the instructions provided on GitHub reproduced the AUROC and AUPRC results to within around 0.001-0.002 of the numbers reported in the paper/the authors' supplementary figures on GitHub.

We renamed ConvNet.py to Basenji.py to make it easier to make the association.

- There is an "FDNABert" and a "DNABert" model available on GitHub but only one DNABert model, I believe, mentioned in the paper.

FDNABert uses the same fine tuning block as FSei based on the embeddings produced by DNABert. DNABert is a simple approach that simply averages over the DNABert embeddings as proposed in the DNABert publication. Both are mentioned in the current version of the manuscript.

- Running FSei with the instructions provided on GitHub did not reproduce the results demonstrated in the paper. In the paper, FSei comes out clearly on top of the pack, with AUROC of 0.815 and AUPRC 0.654. In my run of the code, FSei reported an AUROC of 0.786 and an AUPRC of 0.602.

The command line in GitHub was missing the -p parameter that loads the Sei pre-trained model. This results in training the model from scratch, and hence the worse results.

Errors:
A possibly non-exhaustive list of issues is below; I recommend the code be tested in a fresh environment and the repository updated from there.

We have fixed all the errors and verified the code in a fresh environment as suggested.

1. Only the pip install line for requirements.txt runs (not the conda install line).
2. requirements.txt is missing biopython, transformers, and prettytable packages.

3. “sei.pth” also needs to be copied from the Sei resources; this is not part of the setup instructions.
4. LR.py exits with an sklearn error that originates on LR.py line 127:

“[...]/sklearn/metrics/_ranking.py", line 731:
in _binary_clf_curve
raise ValueError("{0} format is not supported".format(y_type))
ValueError: unknown format is not supported

5. Installing the packages in requirements.txt loads a version of lightgbm (4.0.0) that is not compatible with the code in LGBM.py. LGBM.py passes the argument “verbose_eval” to lgb.cv(); this argument to cv() was removed after lightgbm 3.3.5.

6. After rolling the lightgbm version back to 3.3.5 to address issue #5 above, LGBM.py still exits with an error:

“LGBM.py line 62, in lgb_eval:
return (np.array(cv_result["auc"])).max() :
KeyError: 'auc'”

7. FDNABert.py exits with an error because it cannot import "train_model" from src.train_utils.py. There is no train_model function in train_utils.py.

Organizational notes:

1. Setup of the pretrained Sei model is a bit unclear, as the user has to comb through the Sei repository to find the pieces of those authors’ instructions that are relevant. Consider including all relevant instructions in the body of the IntronRetention Readme.

We automated the process of downloading the necessary files by including commands for doing that.

2. It would be convenient for the user to be able to replicate the results from the paper simply by following the instructions on GitHub. There are a few instances where additional steps are needed. For instance, the .json files for the LR and LGBM models currently point to an output directory that won’t exist unless the user knows to create it when running Preprocess_data_to_numpy.py, and that is not mentioned in the preprocessing instructions.

We have simplified the process as suggested.

3. It would be helpful for the names of the .py files to match the names of the models in the paper (for instance, in the case of the Bassenji-like model).

Done.

4. I believe that “IntronRetention/data/final_data.csv” is the dataset referenced in Section 4, Table 1. Consider clarifying this in Section 4.

We added the following statement in section 4:
The resulting dataset is available in the GitHub repository at \texttt{data/final_data.csv}; reverse complementation is performed while the data is loaded.

Reviewer 2:

- Well written introduction describing current state but deep learning approaches for intron retention prediction have not been mentioned. I do see a few when I search for it. Even if not relevant to current method, might be worth introducing it and comparing and contrasting.

We have added a section in the introduction on deep learning for splicing prediction in which we compare our approach with existing studies, most of them in the context of exon skipping, which has been the primary focus in the research community on splicing.

- line 106 - Sound choice of using SEI framework but perhaps an explanation of the choice of another convolutional layer with pooling and dense layer being added could have been added. In the methods section a mention is made about it being like Enformer model but that uses transformer blocks as well. (7 convolutional blocks with pooling, 11 transformer blocks, and a cropping layer)

The convolutional layer on top of the Sei embeddings was added for the improved accuracy it provided over the approach of simply replacing the classification block. This is now discussed in greater detail. In our approach we only use the pooling layer of the Enformer, which does not use transformer blocks.

- Abstract typo - "DNA langauage model DNABERT-2" line 21

Fixed.
